# Environmental factors and microbial interactions drive microbial community succession during solid-state fermentation of corn husk for microbial biomass protein production

**DOI:** 10.3389/fmicb.2025.1646555

**Published:** 2025-08-18

**Authors:** Yaping Yan, Yinan Sun, Jinna Cui, Junjie Gao, Yingnan Chai, Zhanying Liu

**Affiliations:** ^1^Engineering Research Center of Inner Mongolia for Green Manufacturing in Bio-Fermentation Industry, Hohhot, China; ^2^Specialized Technology Research and Pilot Public Service Platform for Biological Fermentation in Inner Mongolia, Hohhot, China; ^3^Center for Energy Conservation and Emission Reduction in Fermentation Industry in Inner Mongolia, Hohhot, China; ^4^College of Chemical Engineering, Inner Mongolia University of Technology, Hohhot, China; ^5^Inner Mongolia Ecological Environment Scientific Research Institute Limited, Hohhot, China

**Keywords:** corn husk, solid-state fermentation, capillary water, engineered microbial consortium, microbial community succession

## Abstract

Corn husk, a predominant byproduct derived from intensive corn processing, is characterized by high cellulose content, low protein content, and poor palatability, which makes it difficult to be fully utilized by ruminants. This investigation employed corn husk as substrate for microbial protein production through a two-stage open solid-state fermentation (SSF) system using *Aspergillus niger* and yeast strains. The fermentation process yielded a 65.12% enhancement in true protein content. Analysis of microbial community succession dynamics and their regulatory determinants revealed critical correlations with microbial protein production efficiency. Random forest analysis combined with co-occurrence network modeling revealed distinct microbial community dynamics across fermentation phases. During the initial phase (P1), *Bacillus* and *Aspergillus* dominated the community, with their core modules significantly influenced by capillary water, free water, and pH. In the later phase (P2), Saccharomyces and Cyberlindnera took over as dominant genera, primarily shaped by capillary and free water. The constructed microbial consortium comprising *Aspergillus*, *Saccharomyces*, and *Cyberlindnera* exhibited multifactorial regulation involving temperature, pH, capillary water, and free water, along with complex interspecies interactions with members of Firmicutes and Proteobacteria. These findings provide valuable guidance for targeted manipulation of microbial community succession during corn husk fermentation and optimization strategies for microbial biomass protein.

## 1 Introduction

The Food and Agriculture Organization of the United Nations (FAO) projections indicate a global population expansion to 9.7 billion by 2050, accompanied by a 30–50% surge in dietary protein requirements totaling 265 million metric tons (Clark et al., 2020). This demographic growth precipitates a critical imbalance between escalating nutritional demands and constrained protein availability. Microbial protein (MP) has emerged as a viable alternative protein source due to its demonstrated safety profile and production efficiency, positioning it as a strategic solution for addressing this impending nutritional deficit (Carranza-Méndez et al., 2022). Maize (*Zea mays* L.) represents the most extensively cultivated cereal crop globally in terms of planted acreage. FAOStat data indicate global corn production has surpassed 1.137 billion tons (Achli et al., 2024), with China output reaching 289 million tons, representing 25.42% of total global production. Approximately 80 million tons of this yield undergo intensive processing, producing approximately 10 million tons of corn husk as a by-product. The management and accumulation of corn husk further consume energy expenditure and water resource utilization. To mitigate these challenges, solid-state fermentation (SSF) has emerged as an innovative strategy for converting high-cellulose, industrially centralized by-products into microbial protein. This method is distinguished by reduced energy requirements, minimal water consumption, and negligible environmental impact.

In conventional microbial biomass protein production employing fibrous substrates, a mixed-strain fermentation strategy is implemented. Cellulose degradation is predominantly mediated by *Bacillus subtilis* and *Aspergillus niger*, which secrete extracellular cellulases, hemicellulases, and proteases to hydrolyze structural polysaccharides, hemicellulose, and macromolecular proteins into glucose, xylose, and small peptides (Yang et al., 2025; Mandari et al., 2020). Microbial biomass proliferation typically utilizes species such as *Saccharomyces cerevisiae*, *Pichia pastoris*, and *Candida utilis*, selected for their low nutrient demands, rapid growth, and high protein content. These strains are widely applied in solid-state fermentation of agricultural and livestock by-products to produce protein-rich microbial feed (Xu et al., 2025; Chen et al., 2025). In addition, lactic acid bacteria (e.g., *Lactiplantibacillus plantarum*) mediate to microbial community regulation through organic acids production, inducing pH of the fermentation substrate. This pH reduction effectively suppresses the proliferation of certain pathogens and fungi (Tang et al., 2024).

Current investigations into microbial protein feed production have primarily concentrated on strain selection and process optimization (Su et al., 2021a; Sun et al., 2024; Zhang and Zhao, 2022), whereas multi-phase fermentation systems and critical regulatory mechanisms governing fermentation dynamics remain limited.

During SSF, the dynamic succession of microbial communities is influenced by multifactorial interactions. Among environmental parameters, particularly thermal variation, exert direct regulation effects on microbial composition. For instance, controlled thermal adjustments in winter-phase soy sauce fermentation induce substantial restructuring of bacterial structure, whereas fungal communities remain relatively stable (Feng et al., 2024). Concurrently, pH declines below 4.5 lead to effective inhibition of *Aspergillus* proliferation, facilitating yeasts dominance (Yang et al., 2025; Chen et al., 2022). Moisture levels further modulate microbial competition dynamics and metabolic direction by affecting substrate porosity, oxygen diffusion, and solubility of nutrients. Notably, molds proliferate significantly at 75% moisture content, while yeast metabolism favors alcohol production at 60% (Du et al., 2025; Li et al., 2022).

The nutritional profile of fermentation systems is fundamentally regulated by both carbon source typology and concentration gradient. Elevated concentrations of reducing sugars exert inhibitory effects on microbial proliferation in the early phase of fermentation, whereas progressive depletion of carbon substrates in intermediate stages frequently triggers acidogenic microbiota to undergo peak metabolic activity (Xu et al., 2021). Microbial interactions exhibit both competitive and cooperative dynamics. This principle is exemplified in Chinese liquor (Baijiu) fermentation, where dual inoculation strategies combining fungal and bacterial consortia demonstrate enhanced bio processes efficiency and accelerated fermentation kinetics (Tan et al., 2022).

Previous studies into key regulatory factors in SSF primarily focused on traditional fermented foods such as Baijiu, soy sauce, and vinegar (Hao et al., 2021; Wang et al., 2023; Zhang et al., 2024), while microbial biomass protein feed systems have received minimal consideration. Moreover, macro-level analyses frequently fail to capture the direct and indirect influences of environmental variables on microbial symbiosis. Consequently, elucidating the mechanistic drivers underlying microbial successional patterns during protein-enriched microbial feed fermentation represents a critical research imperative.

The present investigation was based on a pilot-scale experiment and employed a two-stage fermentation process. High-throughput sequencing technology was used to analyze the dynamic patterns of microbial community succession (MCS) during corn husk fermentation, while random forest machine learning algorithms identified stage-specific biomarkers and succession trajectories. Network analysis elucidated inter-microbial relationships across distinct fermentation phases, with the combined examination of microbial interactions and environmental responses providing mechanistic insights into MCS establishment. These findings establish a theoretical framework for directed manipulation of microbial consortia during protein feed production from lignocellulosic biomass.

## 2 Materials and methods

### 2.1 Microorganisms and raw materials

*Aspergillus niger* (CGMCC 3.17612), *Saccharomyces cerevisiae* (2.5279), and *Candida utilis* (CGMCC 2.2878) were purchased from the China General Microbiological Culture Collection Center (CGMCC). The experimental materials comprised corn husks, ammonium sulfate (70% purity), and soybean meal (45% crude protein content), all procured from Neimenggu Fufeng Biotechnologies Co., Ltd. Supplementary chemical reagents of specified purity grades were acquired from regional suppliers, including: sodium nitrate LR (99%), potassium dihydrogen phosphate LR (99%), potassium chloride LR (99%), magnesium sulfate LR (97%), protein peptone BR (containing ≥12% total nitrogen and ≥2% amino nitrogen), yeast extract BR (≥35% free amino acids), and glucose LR (99%).

### 2.2 Seed culture preparation

The *Aspergillus niger* seed culture medium contained 12% Brix malt extract, 12 g/L sodium nitrate, 3 g/L monopotassium phosphate, 1 g/L potassium chloride, 1 g/L magnesium sulfate, and 4 g/L tryptone. Sterilization was performed via autoclaving at 121°C for 20 min. Shake flask cultivation proceeded at 30°C and 180 rpm for 36 h. Upon achieving spore count reached ≥10^9^ CFU/mL, a 3 L of spore suspension was prepared for scale-up. This seed culture was then transferred to a 100 L fermenter containing the same medium, maintained at 30°C with 180 rpm, pH 5.8, 0.5 v/v*m aeration, and cultivation for 24 h. When the spore concentration again reached ≥10^9^ CFU/mL, a 5% (v/m, dry basis) inoculum was formulated, producing 60 L of fermentation broth for use in the first stage of fermentation.

The yeasts seed culture medium consisted of 20 g/L peptone, 10 g/L yeast extract, and 20 g/L glucose. Sterilization was achieved through autoclaving at 115°C for 20 min. Shake flask cultivation proceeded at 30°C with 180 rpm until reaching an optical density (OD) of ≥1.5, at which point fermentation was terminated. Both *Saccharomyces cerevisiae* and *Candida utilis* underwent separate culturing to generate 5 L of seed liquid per strain, subsequently employed to inoculate 300 L fermenters for scale-up. The same medium was used in both fermenters under the following conditions: 30°C, 180 rpm, uncontrolled pH, and 0.5 v/v*m aeration. Fermentation was discontinued upon reattainment of OD ≥1.5. Following a 5% (v/m, dry basis) inoculation rate, 200 L of yeast fermentation broth was prepared for use in the second stage of fermentation.

### 2.3 Two-stage fermentation of corn husks

In the first-stage fermentation (P1), 1,000 kg of 20-mesh corn husks, 150 kg wheat bran, and 50 kg crushed soybean meal were quantified and loaded into a sterilizable-compatible vessel. A total of 1,000 L of ultrapure water was incorporated, with a rotary sterilization kettle ensuring complete substrate hydration. Steam injection elevated system pressure to 0.2 MPa, followed by 40-min steam treatment. Post-sterilization, the material underwent cooling to 40°C using screw conveyor and inoculated with *Aspergillus niger* fermentation broth inoculation through an automated inoculation device. The inoculated substrate was subsequently transferred to the fermentation tank. The temperature of the fermentation chamber was stabilized above 25°C. After 36-h fermentation, a white fungal mycelial layer appeared on the surface. Outdoor air was introduced to cool and dry the mixture, and fermentation was completed at 48 h.

For the second-stage fermentation (P2), 2,800 kg of 20-mesh corn husks, 100 kg ammonium sulfate, and 50 kg molasses were combined with primary fermentation residues in the bioreactor. A total of 2,000 L of ultrapure water was introduced, followed by homogenization and 12-h static hydration. Subsequently, 200 L each of *Saccharomyces cerevisiae* and *Candida utilis* fermentation broths were aseptically incorporated with uniform mixing. Upon exceeding 35°C, the material was turned manually to reduce the temperature. Post-aeration, the material was covered with plastic sheeting, leaving ventilation openings at both ends, with fermentation proceeding for a total of 96 h.

### 2.4 Illumina MiSeq sequencing

Genomic DNA was conducted with the TIANNAMP Soil DNA Kit following the manufacturer protocols. Microbial communities were explored by high-throughput sequencing on Illumina MiSeq PE250 platform in Shanghai Majorbio Bio-Pharm Technology Co., Ltd. Amplification of the 16S rRNA gene V3–V4 region utilized universal 516F/806R primer for both the bacteria and archaea communities. The obtained clean sequences were clustered into operational taxonomic units (OTUs) with a 97% sequence similarity threshold.

### 2.5 Statistical analysis

Microbial community analysis was executed within the R statistical framework (R version 4.4.0). PCoA was implemented through the vegan package based on Bray–Curtis dissimilarity matrices derived from bacterial or fungal communities. Permutational multivariate analysis of variance (PERMANOVA) was conducted with 999 permutations to assess intergroup differences in microbial composition. Stage-predictive microbial biomarkers were evaluated via randomForest package-driven modeling, with the fermentation stage set as the response variable and the number of trees set to 1,000, to evaluate the importance of each microorganism in stage prediction. The top 15 species ranked by predictive importance in the random forest model were selected for correlation analysis with environmental factors. Log-transformed abundance values underwent graphical representation through heatmap construction using the pheatmap package.

Intra-stage microbial interaction networks were calculated, and species correlation network data were screened based on thresholds of absolute correlation coefficients >0.9 and correlation test *p*-values <0.01. Network visualization was achieved through Gephi software (version 0.10.1), complemented by modular taxonomic distribution patterns illustrated via stacked column graphs. Mantel tests (vegan package implementation) assessed Pearson correlation between microbial network modules and physicochemical parameters. Predictive modeling through randomForest algorithms subsequently determined environmental factor contributions to α-diversity indices and principal coordinate variance (from PCoA).

### 2.6 Analytical methods

Real-time temperature monitoring of fermentation substrates was conducted with temperature sensors. Moisture content of different types was measured using low-field nuclear magnetic resonance (NMR) analysis (He et al., 2019; Xue et al., 2025). pH was determined using a calibrated pH meter. Filter paper activity (FPA) was assayed via the 3,5-dinitrosalicylic acid (DNS) method (Han et al., 2017), where one enzymatic unit (U) corresponds to glucose liberation of 1 μg per minute from substrate at 50°C. The determination methods for true protein are described in Su et al. (2021b). Comprehensive methodological protocols are procured with Supplementary material S1. Triplicate fermentation pits were established for both P1 and P2 phases, with three biological replicates conducted per experimental group.

## 3 Results and discussion

### 3.1 Changes in physicochemical characteristics during two-stage fermentation

**Figure 1:**
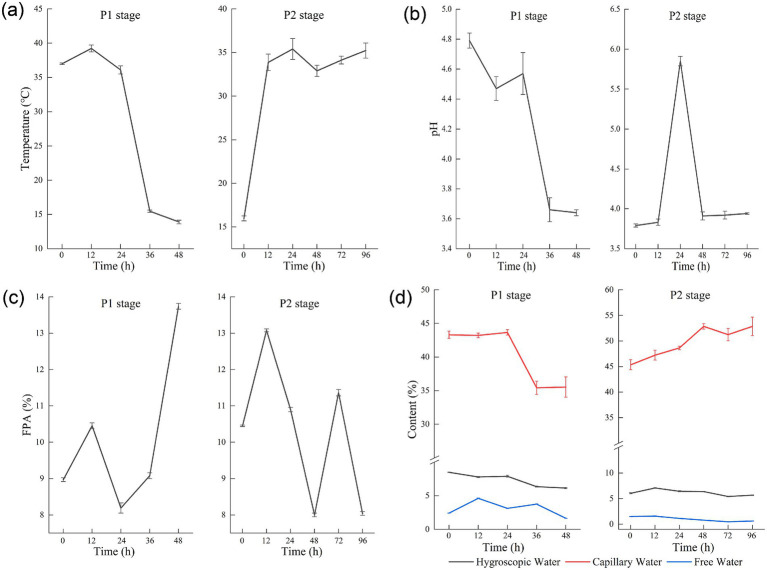
Temperature **(a)**, pH **(b)**, filter paper activity (FPA) **(c)**, and different water forms **(d)** during corn husk fermentation.

Temperature directly impacts microbial growth rate (Zhao et al., 2015). Owing to the poor heat conductivity in solid substrates, temperature control during solid-state fermentation relies on regulating the ambient temperature, ventilation, and manual turning of the substrate. As demonstrated in Figure 1a, substantial metabolic heat production accompanied *Aspergillus niger* proliferation during the P1 stage. At the 12-h fermentation mark, substrate temperatures reached a maximum of 39.23°C. Internal circulation ventilation was implemented to reduce the internal temperatures of the substrate, maintaining operational parameters between 34°C and 35°C.

Mycelia proliferation of *Aspergillus niger* induces hyphal matrix establishment within substrates significantly impeding heat transfer. Manual disruption of the hyphal matrix becomes essential to sustain thermostatic fermentation temperature at 35°C. The P2 fermentation stage initiates under lower thermal conditions, achieving thermal stabilization post-12 h that supports accelerated yeast colonization. The pH value shows an overall decreasing trend during P1, while it slowly rises to 3.94 during P2 (Figure 1b). Primary metabolic activities of *Aspergillus niger* involve preferential carbohydrate catabolism (such as glucose), generating substantial organic acids (citric acid, gluconic acid, oxalic acid) through glycolytic and tricarboxylic acid pathways that drive pH reduction (Latif et al., 2024). Subsequent yeast may use the organic acids accumulated during the *Aspergillus* stage as carbon sources, reducing the acidity of the system (Murata et al., 2021). Proteolytic catabolism and nitrogenous compound degradation (including ammonium sulfate) by yeast populations generate NH₃ through deamination pathways, causing pH to increase (Zhang et al., 2023).

The filter paper enzyme activity (FPA) exhibited a biphasic pattern during P1, characterized by an initial increase, subsequent decline, and final resurgence. Transitioning to P2, a consistent reduction in enzymatic activity was observed (Figure 1c). The transient activity surge at 72 h potentially reflects the inherent heterogeneity of solid-state fermentation substrates. Spatial variations in temperature distribution, moisture content, and oxygen availability across different fermentation layers account for the differential metabolite production (Chen et al., 2024). This heterogeneity was further evidenced by the comparable enzymatic activities measured in the bottom layer samples at 48, 72, and 96 h in Supplementary material S3.

Differential aqueous phase partitioning governs fermentation progression: free water at different fermentation stages, free water hydrodynamic dominance during initial stages facilitates accelerated microbial proliferation, while subsequent phases exhibit capillary water predominance following free water depletion. Capillary and free water content exhibit reduction throughout during P1, with free water levels demonstrating significantly diminishment during P2. Periodic nitrogen supplementation through ammonium sulfate during agitation induces initial capillary water elevation followed by stabilization (Figure 1d). At the end of fermentation, capillary water accounts for nearly 90% of the total water content.

### 3.2 Microbial community dynamics

Progressive modifications occurred in microbial diversity indices during the solid-state fermentation of corn husks. Alpha diversity analysis revealed bacterial communities at P1 stage possessed significantly elevated Chao1 indices relative to P2 stage, though Shannon index values remained comparable between stages. Fungal communities conversely demonstrated increased Chao1 and Shannon indices during P2 compared to P1, indicating substantial fungal community restructuring throughout fermentation compared to relatively conserved bacterial composition (Supplementary Figure S2). This disparity likely originates from artificial inocula-mediated selective enrichment processes, wherein introduced microbial consortia suppress indigenous community while preserving functional equilibrium (Zhang et al., 2025).

**Figure 2:**
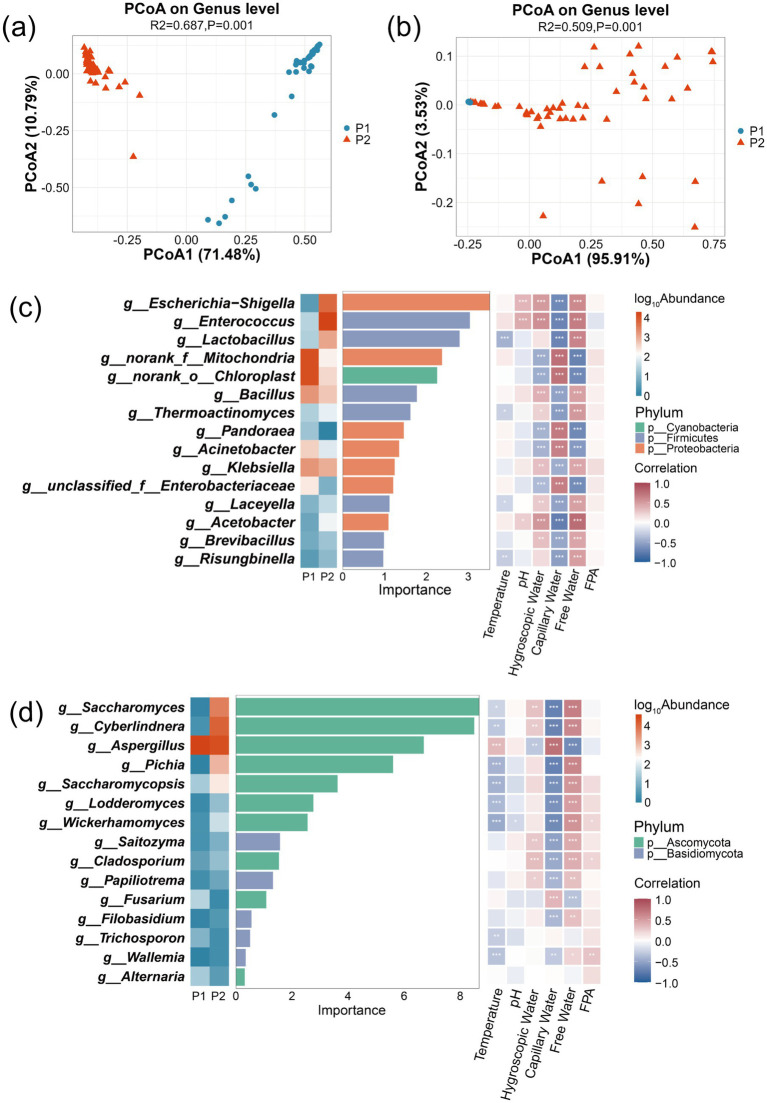
Detection of microbial community structure and biomarker driving MCS during corn husk fermentation using random forest algorithm and PCoA. PCoA of bacterial community structure **(a)** and fungal community structure **(b)**. Fifteen bacterial genera **(c)** and fifteen fungal genera **(d)** discriminating between P1 and P2 stages.

PCoA was implemented to evaluate temporal dynamics in microbial community composition during the staged fermentation of corn husks. Results revealed significant differences in bacterial community structure (PERMANOVA, *R*^2^ = 0.687, *p* = 0.001, Figure 2a) and fungal community structure (PERMANOVA, *R*^2^ = 0.509, *p* = 0.001, Figure 2b) across different fermentation stages. The elevated bacterial *R*^2^ value implies fermentation stages progression accounts for greater variance proportion in bacterial communities, potentially reflecting amplified artificial intervention efficacy during stage transitions. Conversely, fungi communities showed reduced phase-associated variability, indicating oxygen concentration, metabolic products, or stochastic mechanisms exert greater regulatory influence.

To establish associations between fermentation stages and microbial community composition, a random forest algorithm was employed to construct a fermentation stage–microbe association model, identifying 30 key biomarkers (15 bacterial and 15 fungal genera). Taxonomic significance and functional specificity of these biomarkers elucidated stage-specific microbial succession patterns inherent to the biphasic fermentation system. Bacterial biomarkers predominantly comprised enzyme-producing Firmicutes (e.g., *Bacillus* spp.) and metabolically diverse Proteobacteria (Figure 2c), while fungal biomarkers were dominated by Ascomycota members specializing in lignocellulose decomposition and protein biosynthesis, including *Aspergillus*, *Saccharomyces*, and *Cyberlindnera* (Figure 2d).

During P1 stage, Bacillus (6.12% relative abundance) and *Aspergillus* (99.66% relative abundance) synergistically degraded the lignocellulosic complex in corn husks through cellulases and exoglucanase secretion. Upon entering the P2 stage, the environmental pH dropped below 4.0, triggering a functional shift in the community. Notable microbial redistribution occurred, with *Lactobacillus* proportion elevating to 3.56%, supplemented by proliferation of inoculated *Saccharomyces cerevisiae* (12.84%) and *Cyberlindnera* (28.43%), concurrent with *Aspergillus* reduction to 54.25% dominance. This transition established protein-centric microbial succession. Multifactorial drivers underpinned this succession: acidic conditions directly suppressed *Aspergillus* sporulation, whereas soluble saccharides sustained proteogenic microbial metabolism. Consequently, true protein content increased by up to 65.12% (Supplementary Figure S3) (Ma et al., 2022; Ahmad et al., 2022).

The right-side legend displays, from top to bottom, the log-transformed abundance, phylum affiliation, and correlation with environmental factors. Microbial-environmental interactions are annotated using Spearman correlation coefficients with significance thresholds (^*^p < 0.05, ^**^p < 0.01, and ^***^p < 0.001).

### 3.3 Influence of environmental factors on microbial communities

**Figure 3:**
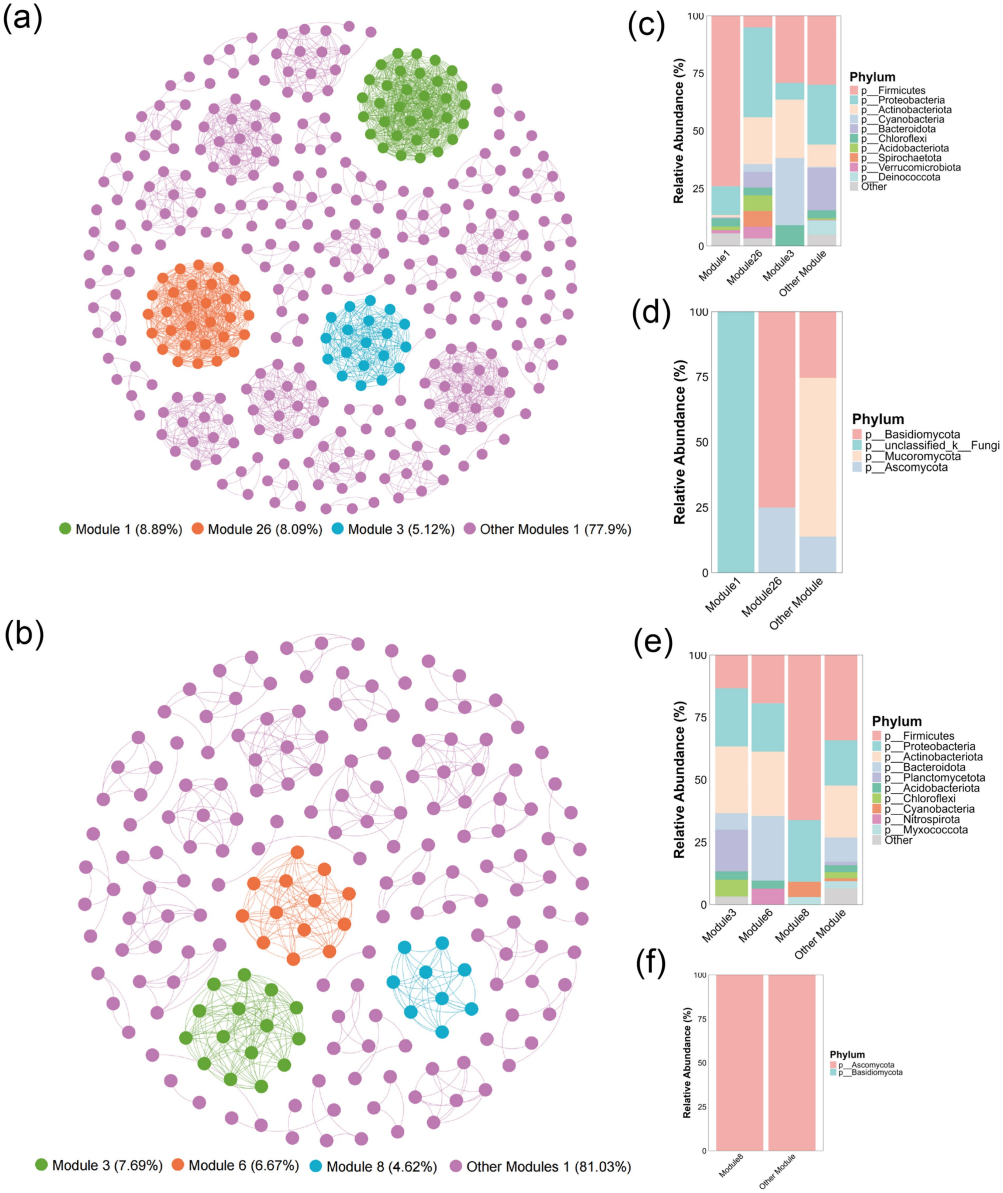
Co-occurrence network analysis (*r* > 0.6, *p* < 0.05) of corn husk fermentation microbiota across two stages, with modular microbial composition profiles. **(a)** P1 stage co-occurrence network; bacterial **(c)** and fungal **(d)** community structures within P1 stage modules. **(b)** P2 stage co-occurrence network; bacterial **(e)** and fungal **(f)** community structures within P2 stage modules.

Symbiotic network analysis serves as an essential methodology for investigating structural and interactive attributes of microbial communities (Wei et al., 2025). This investigation employed genus-level co-occurrence network construction to examine microbial communities structure and environmental parameter associations across pivotal corn husk solid-state fermentation stages (P1 and P2). Network topology analysis revealed 61 modules structures within the P1-stage microbial community, with modules 1 (8.89%), 26 (8.09%), and 3 (5.12%) representing predominant components that collectively established the network core (Figure 3a). Analysis of Module 1 identified 33 genera, predominantly classified under phyla Firmicutes, Proteobacteria, Basidiomycota, unclassified Fungi, and Ascomycota. Modules 26 and 3 consisted of 30 and 19 genera respectively, demonstrating distinct phylum-level taxonomic profiles (Figures 3c,d). Notably, these principal modules displayed elevated intra-modular connectivity densities contrasted by limited inter-modular linkages, constituting a characteristic small-world network configuration. To investigate structural relationships between microbial community structure and environmental factors, Mantel tests were implemented to evaluate module-environmental parameter associations. The results showed that Module 1 exhibited significant positive correlation with capillary water (*r* = 0.41, *p* < 0.01), free water (*r* = 0.21, *p* < 0.05), and pH (*r* = 0.20, *p* < 0.01). Similar correlations were observed for Module 3 with capillary water (*r* = 0.32, *p* < 0.05) and pH (*r* = 0.19, *p* < 0.01) (Supplementary Figure S4a). These observations indicate critical regulatory roles of capillary water, free water, and pH play in regulating microbial community structure and driving modular network assembly.

The P2 stage microbial co-occurrence network comprised 47 modules, with Modules 3 (7.69%), 6 (6.67%), and 8 (4.62%) representing dominant components that collectively formed the core network core structure (Figure 3b). Analysis of Module 3 showed it comprised 15 bacterial genera spanning Firmicutes, Proteobacteria, Actinobacteriota, and Planctomycetota phyla. Modules 6 and 8 contained 13 and 9 genera, respectively. Among these three modules, only Module 8 contained fungal genera (Piniphoma, Sarocladium, and Ustilaginoidea) with overall community abundance below 0.001 (Figures 3e,f). Based on the co-occurrence network analysis, Mantel tests were conducted to explore the correlations between each module at stage P2 and various environmental factors. Module 3 exclusively exhibited significant positive correlation with filter paper activity (FPA) (*r* = 0.13, *p* < 0.05) (Supplementary Figure S4b), suggesting cellulase secretion by Firmicutes and Planctomycetota may drive substrate degradation, with the resultant reducing sugars potentially reinforcing symbiotic interactions within the module (Zhao et al., 2024). Module 8 displayed no significant correlations with environmental factors.

Spearman correlation analysis (Figures 2c,d) between biomarkers and environmental factors demonstrated significant negative correlations of inoculated *Saccharomyces* and *Cyberlindnera* were with capillary water (*p* < 0.001), alongside positive associations with free water (*p* < 0.001). On the contrary, *Aspergillus* had significant positive associations with capillary water (*p* < 0.001) but negative correlations with free water (*p* < 0.001). These differential free water contributions may derive from its role in forming continuous liquid phase that accelerate dissolved carbon source transport and amplify yeast respiratory metabolism via enhanced oxygen solubility, coinciding with carbon flowing towards biomass synthesis (Olivieri et al., 2013). The observed positive correlation between *Aspergillus* and capillary water may originate from hyphal tip secretion of hydrophobins that mediate directional extension through substrate micropores via capillary action. This mechanism enables extracellular enzyme migration along hydrophobin-coated pathways to solid–liquid interfaces, whereas excessive free water reduces hyphal-substrate contact angles (Aimanianda et al., 2009). These findings collectively indicate that strategic regulation of environmental parameters, particularly capillary water and free water balance during stage P2, critically governs cellulose depolymerization efficiency and microbial protein biosynthesis.

### 3.4 Analysis of driving factors for microbial community shift

To investigate the driving forces behind microbial interactions influencing MCS during corn husk fermentation, Mantel tests were used to assess the relationships between biomarkers and other microorganisms within the community. As shown in Figure S5, changes in the abundance of fungal markers alone demonstrated a potential induction of corresponding changes in bacterial abundances, with a correlation coefficient of 0.19. No significant associations were found for other comparisons.

**Figure 4:**
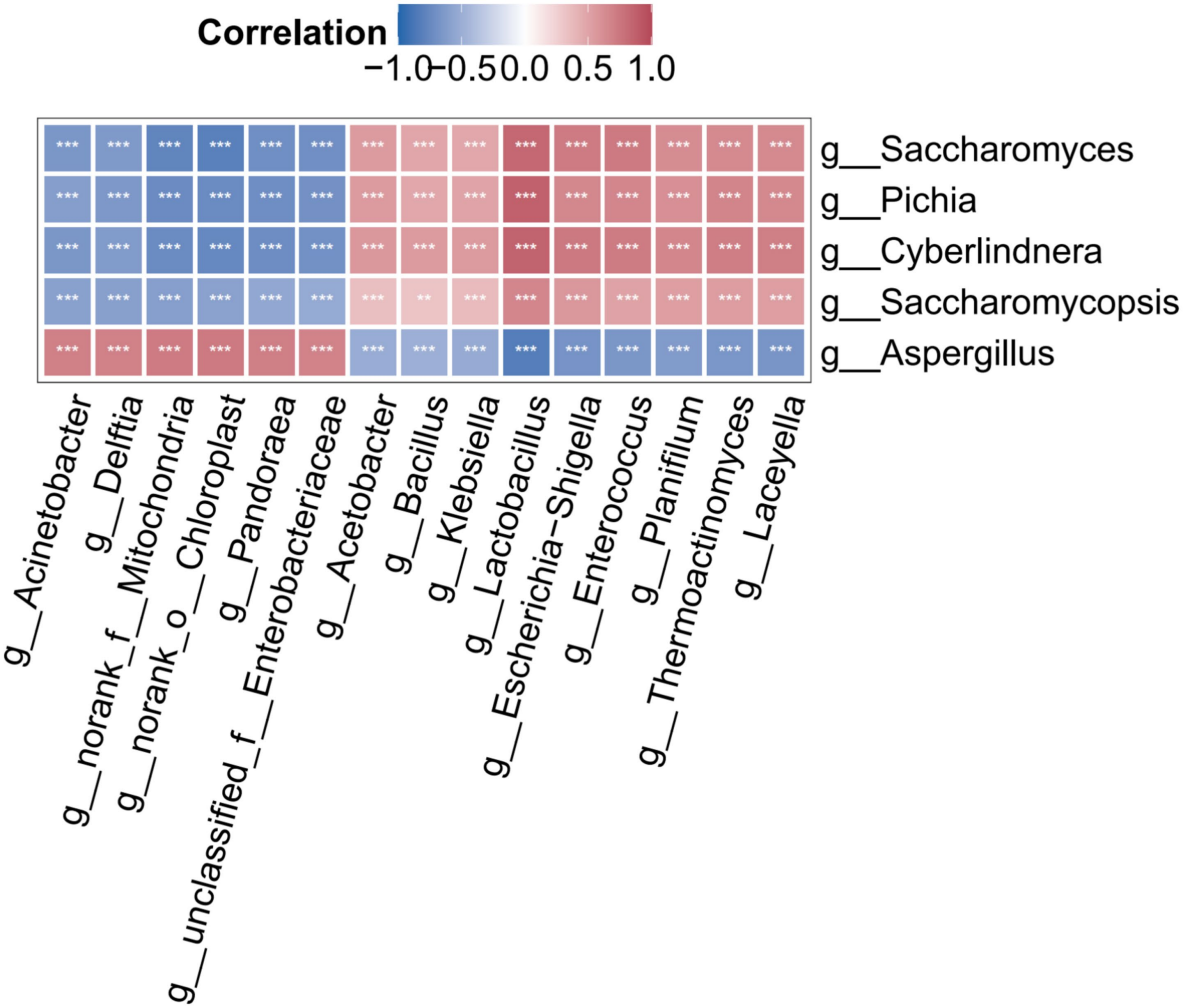
Interactions between fungal and bacterial biomarker microorganisms.

To investigate interactions among microbial biomarkers, correlation analyses between the predominant fungal genera (*Saccharomyces*, *Cyberlindnera*, *Aspergillus*, *Pichia*, and *Saccharomycopsis*; Figure 2d) and primary bacterial biomarkers (top 15) were performed (Figure 4). Mantel tests and network topology revealed significant positive associations between yeast genera (*Saccharomyces*, *Cyberlindnera*, etc.) and Firmicutes (such as lactic acid bacteria and *Bacillus* species). Metabolic activity of these yeasts facilitated soluble sugar release and environmental pH, synergistically promoting the proliferation of cellulolytic bacteria and probiotics, thereby establishing a competitive-symbiotic nutritional network.

In contrast, yeast populations exhibited marked negative correlations with Proteobacteria representatives (e.g., *Acinetobacter*), indicating environmental acidification during late fermentation phase prevents proliferation of these non-dominant bacteria taxa. Notably, *Aspergillus* demonstrated pronounced positive correlations with multiple Proteobacteria biomarkers throughout phase P1, attributable to shared resource utilization capabilities and environmental adaptability. Secretion of cellulases, proteases, and hydrolytic enzymes by *Aspergillus* facilitates macromolecular organics compound degradation, generating metabolic substrates for Proteobacteria saprophytes (e.g., *Acinetobacter*), thereby establishing metabolic symbiosis. They both preferred microaerobic habitat as indicated by the microaerophiles label, and possessed complementary functions (e.g., ability to degrade complex carbon sources) that formed a stable early community (Zhou et al., 2023).

However, upon progression to fermentation stage P2, rapid proliferation of acidogenic taxa such as lactic acid bacteria induced marked pH reduction, concurrent with coordinated population declines in *Aspergillus* and *Proteobacteria* populations attributable to their limited acid tolerance. During this phase, dominance of the yeast-Firmicutes consortium emerged through niche specialization and antimicrobial compound biosynthesis, conferring community stability (Noé Aguilar et al., 2001). The initial positive correlation fundamentally reflects metabolic coordination and environmental co-adaptation within early-stage fermentative microbiota, while parallel population reductions illustrate environmental filtration against these non-adaptive taxa. These findings establish a theoretical framework for accelerated elimination of non-dominant bacteria taxa and enhancement of synthetic microbial consortia through targeted community modulation during initial fermentation stage.

### 3.5 Analysis of potential driving factors for microbial community succession

**Figure 5:**
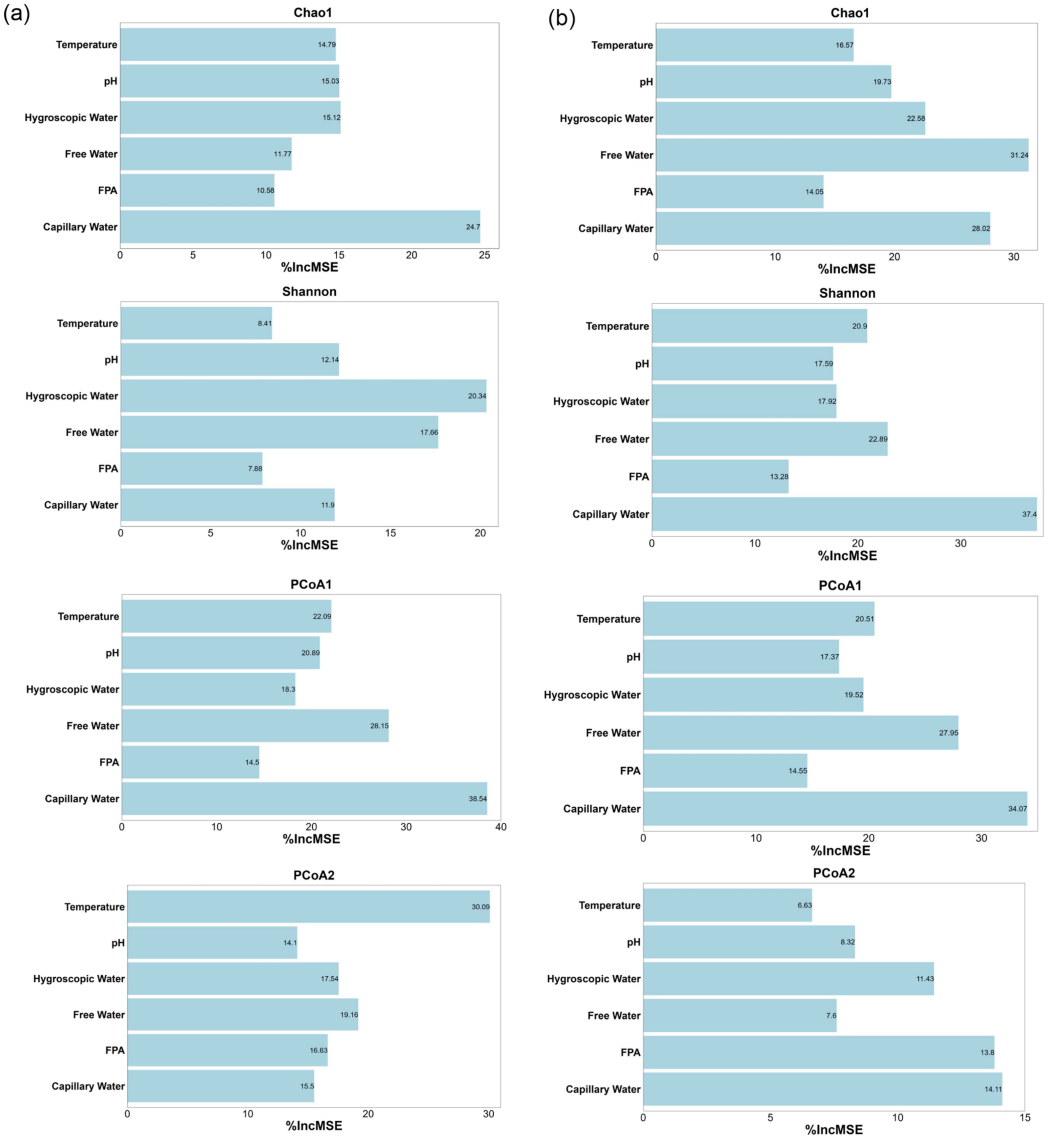
Importance of environmental factors driving the succession of bacterial **(a)** and fungal **(b)** communities during corn husk fermentation.

Random forest models were implemented to identify determinants of microbial community assembly dynamics during corn husk fermentation. As illustrated in Figures 5a,b, capillary water emerged as the predominant ecological determinant of structural divergence in both bacterial and fungal community.

For bacterial assemblages, capillary water exerted significant impacted influence on taxonomic richness (Chao1 index) and explained 24% of variance along the PCoA1. This divergence corresponds to capillary water-mediated oxygen gradients within substrate pore networks, which regulate diffusion kinetics of soluble metabolites. Such physicochemical modulation enhanced niche diversification within cellulolytic bacterial lineages, particularly Firmicutes. Hygroscopic water mediated regulation of the Shannon diversity index through water activity (Aw)-dependent mechanisms, lowering microbial osmotic pressure and accounting for 20% of observed variance. Temperature emerged as the primary determinant of variation along the PCoA2, with thermodynamic regulation mechanisms explaining 30% of system variance.

In fungal community, capillary water demonstrated significant contributions exceeding 28% both Shannon index and PCoA1 variation, while free water mediated regulation of the Chao1 index by influencing liquid film formation, accounting for 31% of observed variance. Crucially, heterogeneous distribution of capillary water generated localized microenvironments gradients, including oxygen partial pressure fluctuations and pH variations, enabling selective dominance of specific microbial taxa. Concurrently, this physicochemical parameter orchestrated nutrient mobilization and microbial synergistic interactions, exemplified by complementary metabolic pathways, thereby ensuring functional equilibrium of the fermentation ecosystem (Chen et al., 2023).

These results establish a strategic insight for modulating microbial community succession to optimize microbial protein biosynthesis efficiency in corn husk fermentation systems.

## 4 Conclusion

MCS during corn husk solid-state fermentation progresses through two distinct stages, characterized by stage-specific environmental modulation of co-occurrence network module dynamics. Thirty bacterial genera were identified as MCS biomarkers, with succession patterns of core inoculated microbial taxa governed by synergistic interactions between abiotic parameters and biomarker activity. Dynamic spatial partitioning of capillary water and free water emerged as the central regulator of community succession trajectories, exerting significant effects on structural reorganization of bacterial community structure and fungal diversity. These findings provide theoretical guidance for precision engineering of synthetic microbial community succession and optimization of microbial protein biosynthesis in corn husk fermentation.

## Data Availability

The original contributions presented in the study are publicly available. This data can be found here: https://www.ncbi.nlm.nih.gov/bioproject//PRJNA1303323.
