# Supplementary material for: Environmental factors and microbial interactions drive microbial community succession during solid-state fermentation of corn husk for microbial biomass protein production
Source: Front Microbiol. 2025 Aug 18;16:1646555. doi: 10.3389/fmicb.2025.1646555 (PMC12399522; doi:10.3389/fmicb.2025.1646555)
Supplement: Supplementary file 6 [file Data_Sheet_6.pdf]

Supplementary data 1: Sampling method

1. First stage fermentation

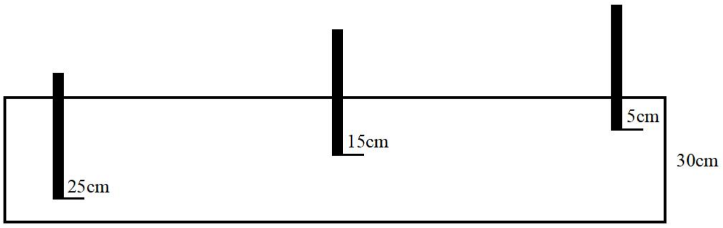

2. Second stage fermentation

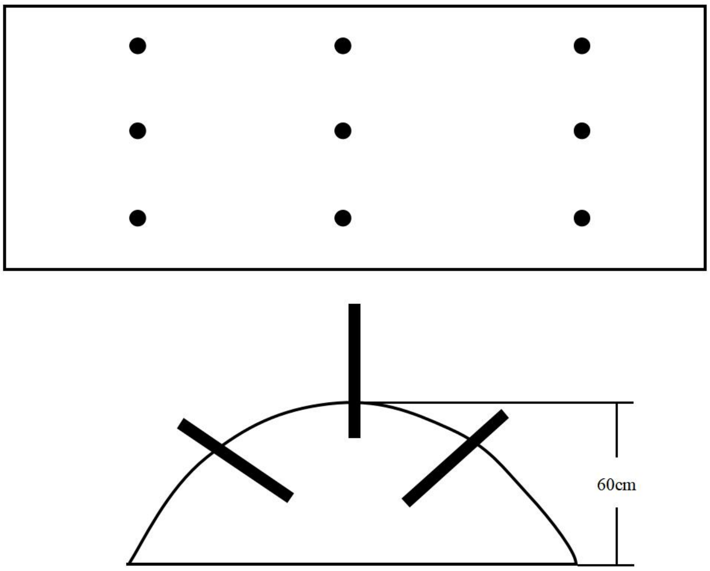

3. Contents of different forms of water measured by low-field NMR and MRI

3.1 Analysis method

Measurements of transverse relaxation time were performed using an NMI20-Analyst (Niumag Electric Corporation, Shanghai, China). The detection method was as previously described [1]. A standard curve for NMR determination of

water content was first prepared, from which a calculation formula was derived and subsequently based on the previously described method for different forms of water [2].

3.2 Establishment of standard curve

The relationship between the water volume within the fermentation matrix and low-field nuclear magnetic resonance signal intensity was examined. Figure 3 presents the calibration curve equation y=4324.45x with a coefficient of determination (R^2) of 0.99953, where y denotes signal intensity and x represents water volume. This mathematical relationship demonstrates a robust linear correlation between the moisture content of solid-state fermentation substrates and corresponding low-field nuclear magnetic resonance measurements.

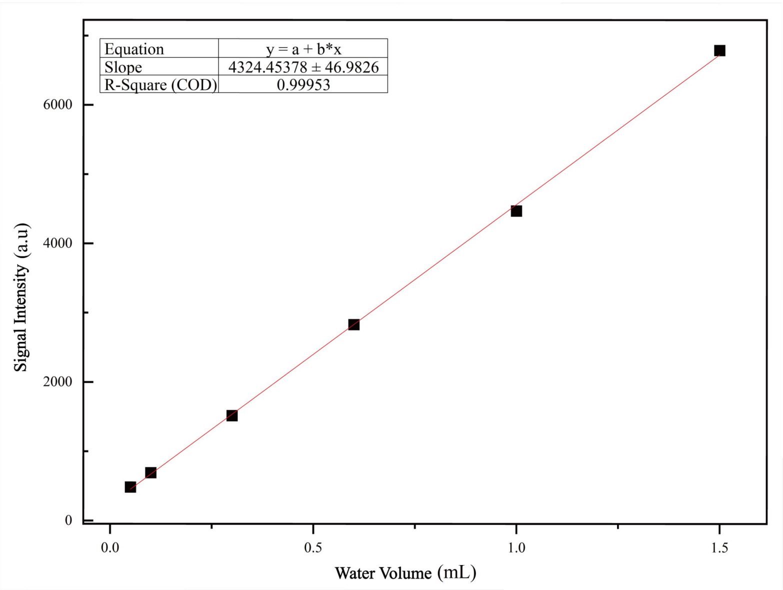

Figure 3 Standard curve of moisture content by low-field nuclear magnetic resonance

References：

[1] Wang, Y.H., Hu, S.S., Yang, Z.Y., Jin, Q., Ren, A., He, Q., Yu, H.S., and Zhao, M.W. (2021). Three-phase structure analysis of sawdust cultivation substrates and rapid detection of the optimal water environment for Lentinula edodes growth based on LF-NMR. Cellulose. 28, 6917-6933. https://doi.org/10.1007/s10570-021-03987-4.

[2] He, Q., and Chen, H.Z. (2015). Comparative study on occurrence characteristics

of matrix water in static and gas double-dynamic solid-state fermentations using low-field NMR and MRI. Analytical and Bioanalytical Chemistry. 407, 115-9123. https://doi.org/10.1007/s00216-015-9077-4.
