# Supplementary material for: Environmental factors and microbial interactions drive microbial community succession during solid-state fermentation of corn husk for microbial biomass protein production
Source: Front Microbiol. 2025 Aug 18;16:1646555. doi: 10.3389/fmicb.2025.1646555 (PMC12399522; doi:10.3389/fmicb.2025.1646555)
Supplement: Supplementary file 7 [file Data_Sheet_7.pdf]

Table 1 FPA of different sampling layers in P2 stage

P2 stage	FPA U/mL		
	Upper	Middle	Under
0	9.37	8.27	13.72
12	8.55	17.13	13.56
24	10.35	13.21	9.15
48	9.81	6.94	7.24
72	13.35	12.92	7.8
96	9.49	6.4	8.23

Table 2 True protein content of different sampling layers in P2 stage

P2 stage	True protein %		
	Upper	Middle	Under
0	7.54	8.21	8.36
12	10.66	10.5	9.95
24	10.38	11.59	10.62
48	11.89	11.44	10.37
72	12.06	11.54	11.62
96	12.45	12.48	12.99
Growth Rate %	65.12	52.01	55.38
