# Supplementary figures and images for: Environmental factors and microbial interactions drive microbial community succession during solid-state fermentation of corn husk for microbial biomass protein production
Source: Front Microbiol. 2025 Aug 18;16:1646555. doi: 10.3389/fmicb.2025.1646555 (PMC12399522; doi:10.3389/fmicb.2025.1646555)

Supplementary data 2: Alpha diversity analysis

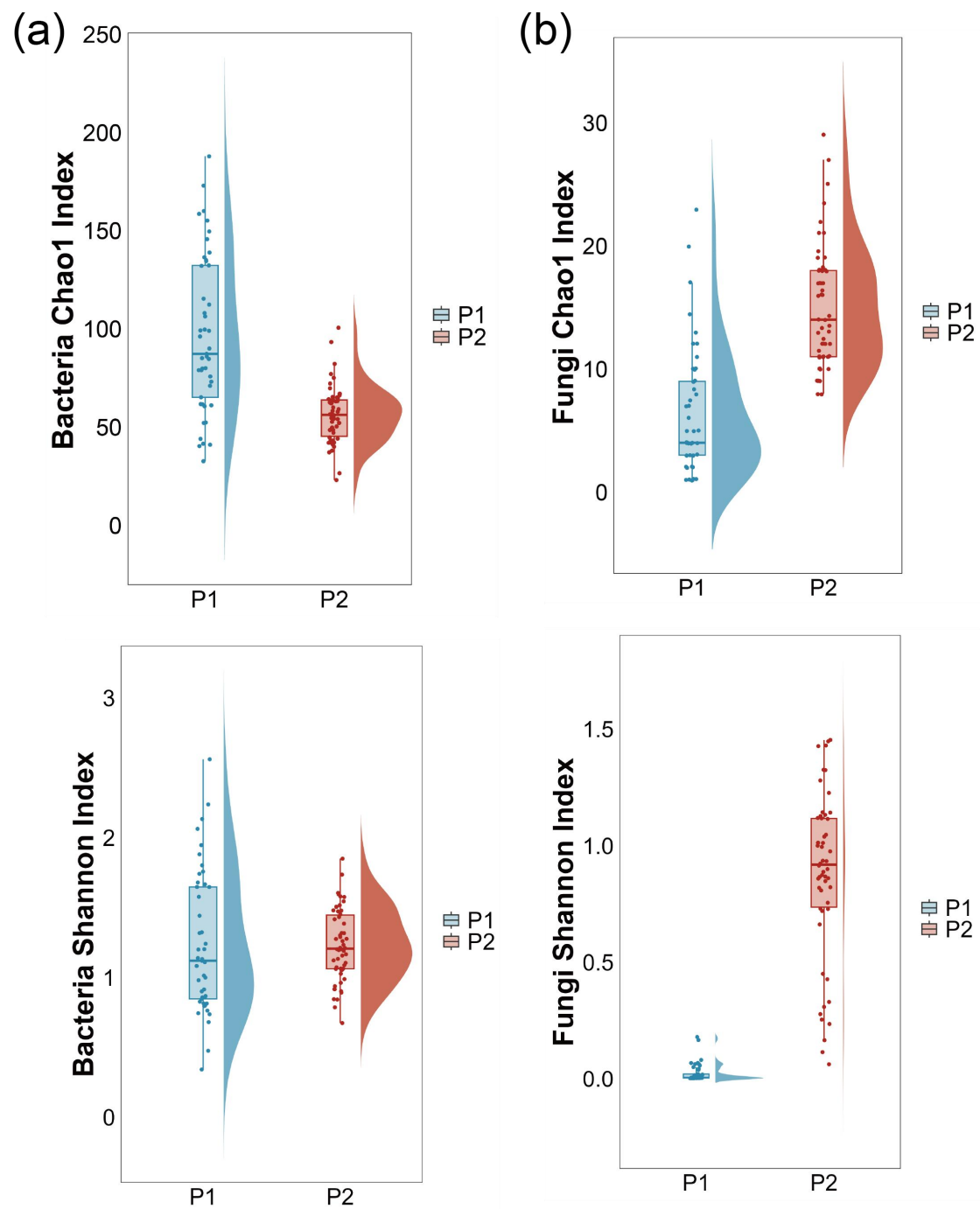

Supplement: Supplementary file 8 [file Image_1.pdf]
