# Supplementary material for: Environmental factors and microbial interactions drive microbial community succession during solid-state fermentation of corn husk for microbial biomass protein production
Source: Front Microbiol. 2025 Aug 18;16:1646555. doi: 10.3389/fmicb.2025.1646555 (PMC12399522; doi:10.3389/fmicb.2025.1646555)

Supplementary data 4: shows the correlation between each module and environmental factors in the P1 stage, as well as the correlation between each module and environmental factors in the P2 stage. The line color represents the p-value level, the line thickness represents the correlation size, and the block color represents the Pearson correlation size of environmental factors. The pairwise correlation between bacterial community module and environmental factor (a), as well as the pairwise correlation between fungal community module and environmental factor (b)

(a)

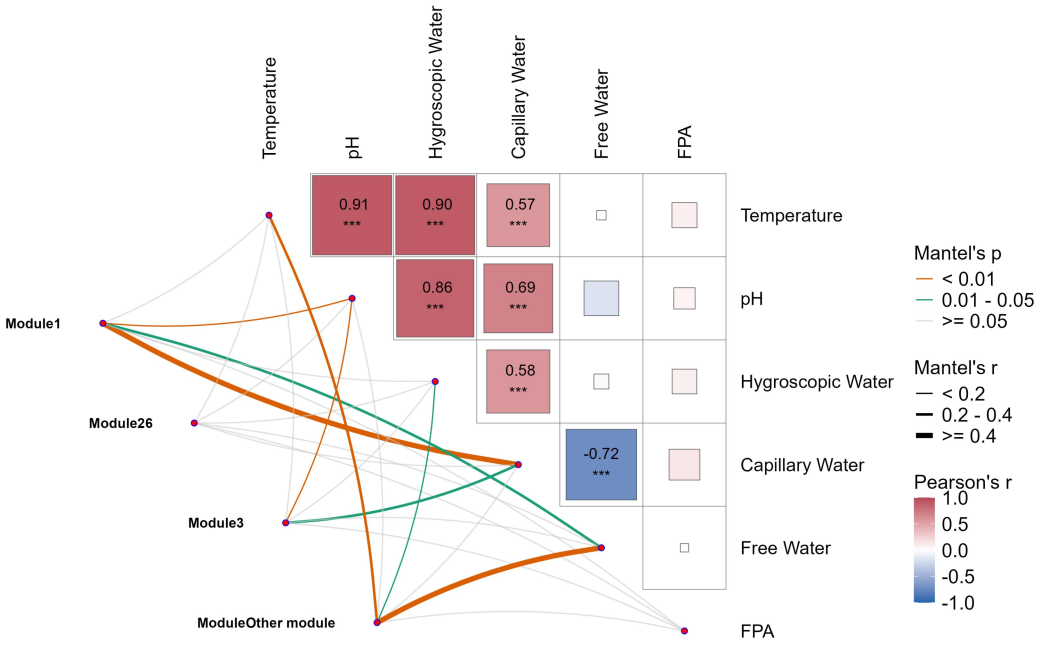

(b)

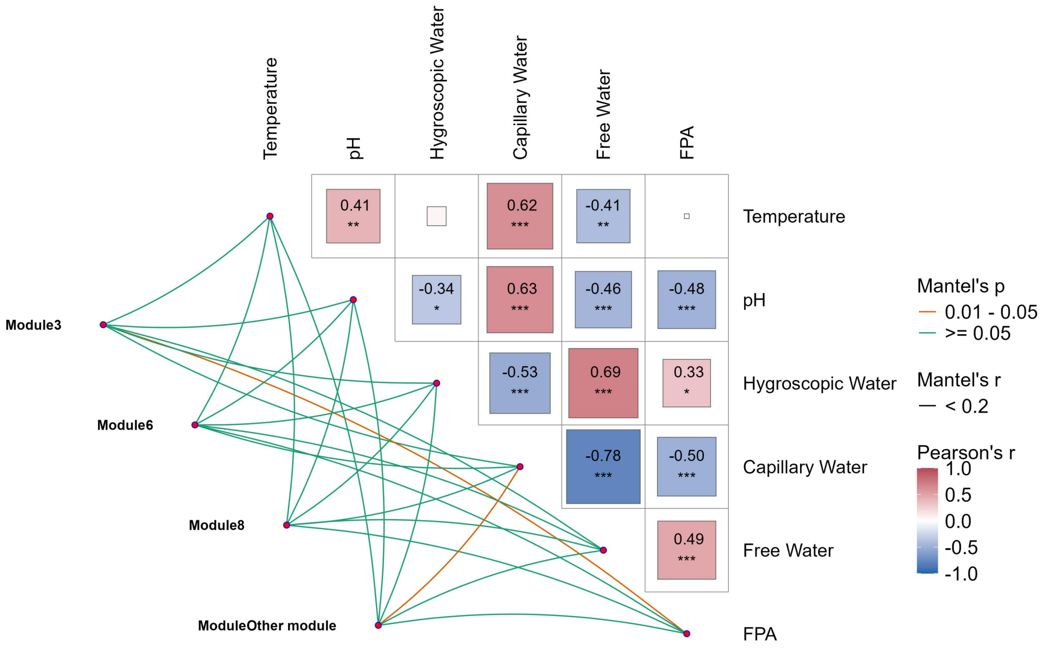

Supplement: Supplementary file 9 [file Image_2.pdf]
